# Two human antibodies to a meningococcal serogroup B vaccine antigen enhance binding of complement Factor H by stabilizing the Factor H binding site

**DOI:** 10.1371/journal.ppat.1009655

**Published:** 2021-06-14

**Authors:** Nathaniel A. Sands, Peter T. Beernink

**Affiliations:** Division of Infectious Diseases and Global Health, Department of Pediatrics, School of Medicine, University of California San Francisco, San Francisco, California, United States of America; Simon Fraser University, CANADA

## Abstract

Microbial pathogens bind host complement regulatory proteins to evade the immune system. The bacterial pathogen *Neisseria meningitidis*, or meningococcus, binds several complement regulators, including human Factor H (FH). FH binding protein (FHbp) is a component of two licensed meningococcal vaccines and in mice FHbp elicits antibodies that inhibit binding of FH to FHbp, which defeat the bacterial evasion mechanism. However, humans vaccinated with FHbp develop antibodies that enhance binding of FH to the bacteria, which could limit the effectiveness of the vaccines. In the present study, we show that two vaccine-elicited antibody fragments (Fabs) isolated from different human subjects increase binding of complement FH to meningococcal FHbp by ELISA. The two Fabs have different effects on the kinetics of FH binding to immobilized FHbp as measured by surface plasmon resonance. The 1.7- and 2.0-Å resolution X-ray crystal structures of the Fabs in complexes with FHbp illustrate that the two Fabs bind to similar epitopes on the amino-terminal domain of FHbp, adjacent to the FH binding site. Superposition models of ternary complexes of each Fab with FHbp and FH show that there is likely minimal contact between the Fabs and FH. Collectively, the structures reveal that the Fabs enhance binding of FH to FHbp by altering the conformations and mobilities of two loops adjacent to the FH binding site of FHbp. In addition, the 1.5 Å-resolution structure of one of the isolated Fabs defines the structural rearrangements associated with binding to FHbp. The FH-enhancing human Fabs, which are mirrored in the human polyclonal antibody responses, have important implications for tuning the effectiveness of FHbp-based vaccines.

## Author summary

Microbial pathogens bind specific host proteins to invade the host or evade host immunity. The bacterial pathogen meningococcus causes sepsis and meningitis, and binds a protein in the human immune system known as Factor H (FH). FH binding protein (FHbp) is a component of two licensed vaccines and can produce antibodies that block this evasion mechanism. Surprisingly, humans vaccinated with FHbp develop antibodies that enhance FH binding. To understand the biological mechanism, here we show that two human antibody fragments (Fabs) increase binding of human FH to FHbp by changing the binding kinetics. The three-dimensional structures of the two Fabs bound to FHbp reveal that the Fabs enhance FH binding through structural rearrangement in a region of FHbp adjacent to the FH binding site. A better understanding of FH enhancement by human Fabs can inform the continued development of meningococcal vaccines by eliminating the binding sites for such antibodies.

## Introduction

Like many microbial pathogens, the bacterium *Neisseria meningitidis*, which is also known as meningococcus, uses surface proteins to recruit soluble complement inhibitors. One of these inhibitors is Factor H (FH), which down-regulates complement activation on the bacterial surface [1,2]. FH binding to the opsonin C3b promotes decay of the alternative pathway (AP) C3 convertase, C3bBb, by competing for the Bb binding site, a process known as decay acceleration activity [3,4]. FH also serves as a cofactor for Factor I to cleave active C3b into inactive iC3b, which prevents the formation of the AP C3 convertase [5,6]. Together, these FH functions effectively block the amplification loop of the AP and limit the generation of C3b, which binds and activates the classical pathway C3 convertase, C4bC2b [7].

Meningococci produce three proteins that function as receptors for human complement FH [8–10]. One of these proteins, which had been independently discovered as GNA1870 and LP2086 [11,12], was found to bind FH and renamed Factor H binding protein (FHbp) [8]. The other two FH receptors are Neisserial surface protein A (NspA) and Porin B2 (PorB2) [9,10,13]. Among the three FH receptors, FHbp is generally considered to be the most important for most meningococcal strains. FHbp specifically binds human and non-human primate FH [14,15], but not rabbit or rodent FH [14,16]. FHbp was developed as a key antigen in two licensed meningococcal serogroup B vaccines [17,18].

Other investigators hypothesized that binding of a host protein decreases the immunogenicity of a microbial vaccine antigen [2]. Subsequently, we and our colleagues showed that human FH transgenic mice produced lower protective antibody responses to FHbp and NspA than did wild-type mice [16,19–21]. In the case of FHbp, the higher protective antibody responses of wild-type mice were associated with the ability of the antibodies to inhibit FH binding, and the lower responses of human FH transgenic mice were associated with the antibodies enhancing FH binding [19].

In a subsequent study, we characterized ten human anti-FHbp Fab fragments from three humans vaccinated with one of the licensed vaccines, MenB-4C [22]. By flow cytometry, four of the Fabs enhanced FH binding to the bacteria by factors of three- to six-fold, five Fabs were neutral with respect to FH binding, and one had ~2-fold inhibition. In the absence of vaccine-elicited antibodies that inhibit FH binding, antibody enhancement of FH binding has the potential to limit vaccine effectiveness by increasing the amount of FH bound to the bacterial surface. Therefore, studies of these antibodies are important for understanding mechanisms of protection by microbial vaccine antigens that interact with host complement proteins. In the present study, we report immunological and biophysical studies of two vaccine-elicited human antibodies that enhance binding of complement FH to the meningococcal virulence factor and vaccine antigen FHbp.

## Results

### Binding of Fabs to FHbp

**Fig 1:**
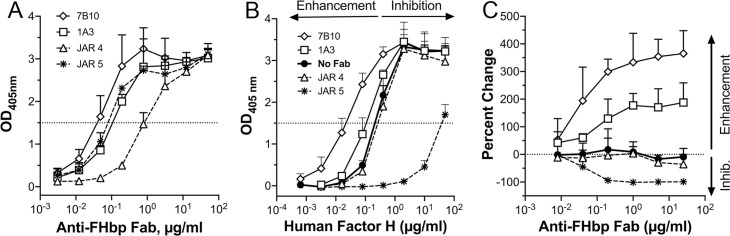
Binding of Fabs to FHbp and enhancement of human FH binding to FHbp by human anti-FHbp Fabs. (A) Direct binding of solution-phase human and chimeric Fabs to immobilized FHbp by ELISA. (B) Enhancement or inhibition of FH binding to FHbp in the presence of a fixed concentration of human and chimeric Fabs. Binding of FH in the absence of Fab is shown by filled circular symbols. Enhancement of FH binding to FHbp is represented by a shift to the left relative to the control without Fab, i.e. a lower FH concentration needed to reach the same optical density (OD_405nm_); inhibition is shown by a shift to the right. (C) Enhancement or inhibition of FH binding in the presence of a fixed concentration of FH. Enhancement is represented by a positive change in binding of FH compared with the control without Fab and inhibition is shown by a negative change. For all three panels, the means and 2 standard errors (SE) of triplicate measurements are shown.

We tested commercially produced lots of two human anti-FHbp Fabs and two control mouse-human chimeric Fabs for concentration-dependent binding to purified FHbp by enzyme-linked immunosorbent assay (ELISA). The human Fabs were two of the four that enhanced binding of FH to FHbp by flow cytometry in our previous study [22]. The control Fabs were derived from two mouse anti-FHbp monoclonal antibodies (MAbs), JAR 5, which inhibits binding of FH to FHbp, and JAR 4, which does not [8,23]. All four Fabs exhibited sigmoidal binding to the nominal FHbp antigen, ID 1 (http://pubmlst.org/neisseria/fHbp), the sequence variant that is in the licensed MenB-4C vaccine and that was used to raise the mouse MAbs JAR 4 and JAR 5 (**Fig 1A**). The two human Fabs and the JAR 5 control Fab gave binding curves with midpoints at similar concentrations, whereas the JAR 4 control Fab had approximately 10-fold lower binding to FHbp.

We further validated the newly produced Fabs by testing their cross-reactivity with different FHbp sequence variants. The four Fabs bound to the three FHbp variants with the highest sequence identity to the nominal antigen, ID 1 (100% identity), ID 13 (93% identity) and ID 14 (91% identity) (**S1 Fig**). In addition, control Fab JAR 4 cross-reacted with FHbp ID 22 (69% identity) and human Fab 7B10 cross-reacted with ID 55 (87% identity). The binding patterns for the control Fabs are consistent with the known locations of the epitopes recognized by these Fabs [24,25], and with the modular architecture of FHbp [26].

### Modulation of binding of FH to FHbp by human anti-FHbp Fabs

Next, we tested the ability of the human Fabs to modulate the binding of human complement FH to FHbp by ELISA using a fixed concentration of Fab and variable concentrations of human FH (**Fig 1B**). Compared to negative control reactions in the absence of Fab (filled circular symbols), the control Fab JAR 4 had no effect on the binding of human FH to FHbp. In contrast, control Fab JAR 5 (asterisks) inhibited FH binding ~100-fold. The two human Fabs showed enhancement of FH binding by approximately two-fold for 1A3 (open squares) and ten-fold for 7B10 (open diamonds). In a converse experiment in which the concentration of FH was constant and that of the Fabs was varied, binding of Fab JAR 4 also had no effect and JAR 5 showed almost complete inhibition of FH binding to FHbp (**Fig 1C**). In this assay, the human Fabs 1A3 and 7B10 enhanced FH binding by ~2.8- and 4.5-fold, respectively. Thus, each of the two human Fabs enhanced binding of FH to purified FHbp, similar to our previous measurements of FH binding to live bacteria [22].

**Fig 2:**
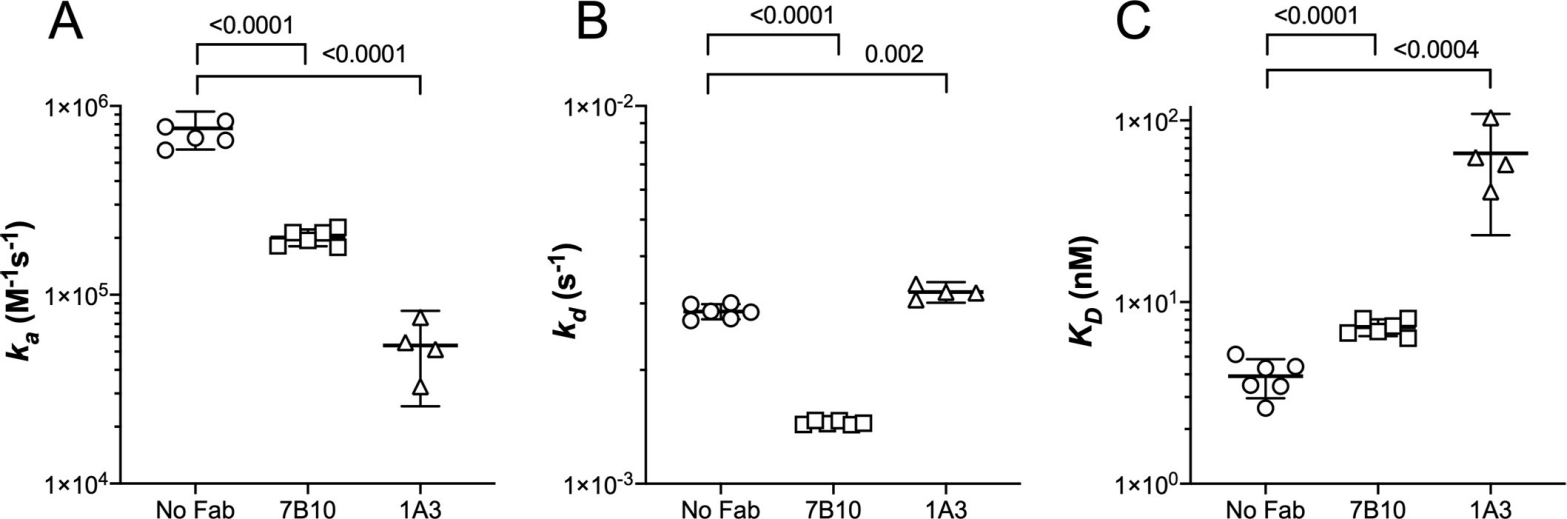
Kinetic parameters of Fab-mediated binding of FH to FHbp determined by surface plasmon resonance. (A) Association rate constant, *k*_*a*_. (B) dissociation rate constant, *k*_*d*_. (C) apparent dissociation constant, *K*_*D*_. For all three panels, the means and 2 SE of four to six replicates are shown. The *p*-values for two-tailed *t*-tests comparing the parameters of FH binding in the presence versus the absence of Fab were calculated with Prism 8.3 (GraphPad).

To further investigate the kinetic effects, we measured binding of different concentrations of solution-phase FH to immobilized FHbp in the absence or presence of each of the human Fabs by surface plasmon resonance (SPR). An increase in Response Units (RU) was observed for binding of FH to FHbp in the presence of the human Fabs, which was additional evidence that these Fabs did not inhibit FH binding (**S2 Fig**). Human Fab 7B10 decreased both the association rate constant (*k*_*a*_) and the dissociation rate constant (*k*_*d*_), which resulted in a slight increase in the apparent dissociation constant (*K*_*D*_) (**Fig 2 and S1 Table**). Human Fab 1A3 also decreased the *k*_*a*_, but had little effect on the *k*_*d*_, and resulted in a ~10-fold increase in *K*_*D*_. Therefore, the two Fabs appeared to alter the FH binding kinetics by different mechanisms.

### Structural bases for effects of Fab binding on FH-FHbp interaction

**Fig 3:**
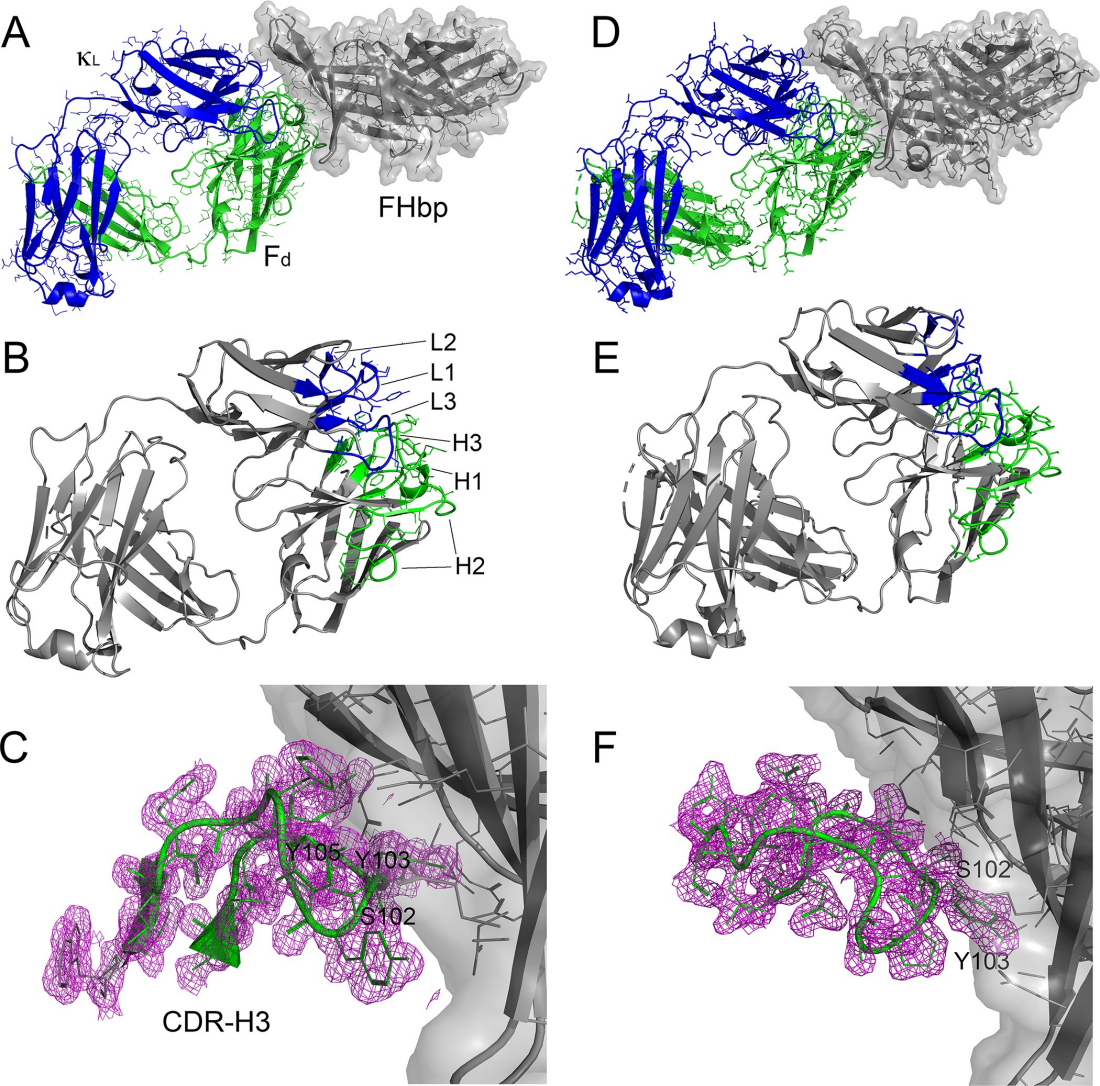
Structures of human Fabs 1A3 and 7B10 bound to FHbp vaccine antigen. (A) Overall view of Fab 1A3-FHbp complex. FHbp is oriented with the Factor H binding site facing toward the top. (B) View of paratope highlighting CDR loops. (C) Electron density map of CDR-H3. The Fab heavy chain is shown in green, the light chain is shown in blue, and FHbp is shown in grey. (D-F) Structure of human Fab 7B10 bound to FHbp vaccine antigen with the same coloring scheme as panels A-C. Figure constructed using PyMol (Schrodinger, LLC).

To determine the structural bases for the complex effects of the two human Fabs on FH binding to FHbp, we crystallized and determined the structures of the two human Fabs in complexes with FHbp by X-ray crystallography. We refined the structures of the Fab 1A3 and 7B10 complexes with FHbp at high-resolution limits of 1.7 and 2.0 Å, respectively; the data collection and refinement statistics are shown in **Table 1**. The overall structures, the complementarity determining region (CDR) loops, and representative electron density maps of the two Fab complexes are presented in **Fig 3**. Despite the differences between the two Fabs in their effects on FH binding and kinetics, and their difference in cross-reactivity with one of the FHbp variants tested, they bound to similar epitopes on the amino-terminal domain of FHbp.

**Table 1:** X-ray data collection and refinement statistics^a^.

Parameter (PDB ID)	Fab 1A3 FHbp Complex (7LCV)	Fab 7B10-FHbp Complex (7KET)	Fab 7B10 (7KE1)
Wavelength	1.116	1.116	1.116
Resolution range (Å)	81.2–1.7 (1.76–1.7)	107.1–2.0 (2.1–2.0)	70.5–1.5 (1.6–1.5)
Space group	P 1	P 1 2_1_ 1	P 1 2_1_ 1
Unit cell lengths (a, b, c)	39.47 52.37 84.56	38.58 86.03 107.89	67.77 97.26 70.47
Unit cell angles (α, β, γ)	73.78 84.48 74.17	90.00 96.91 90.00	90.00 90.37 90.00
Total reflections	234,804 (23,643)	126,296 (10,165)	985,294 (97,061)
Unique reflections	65,915 (6,477)	46,166 (3,877)	143,102 (13,922)
Multiplicity	3.6 (3.6)	2.7 (2.4)	6.9 (7.0)
Completeness (%)	93.06 (93.14)	94.96 (82.44)	98.07 (96.35)
Mean I/sigma(I)	11.59 (1.30)	8.02 (0.53)	12.18 (1.89)
Wilson B-factor	30.88	45.33	18.13
R-merge	0.052 (0.84)	0.101 (1.66)	0.167 (1.01)
R-meas	0.06124 (0.983)	0.123 (2.085)	0.180 (1.086)
R-pim	0.0321 (0.513)	0.0706 (1.241)	0.067 (0.394)
CC^1/2^	0.998 (0.798)	0.311 (0.072)	0.998 (0.702)
CC*	1 (0.942)	0.689 (0.366)	0.999 (0.908)
Reflections used in refinement	64,068 (6437)	44,971 (3,877)	142,917 (13,922)
Reflections used for R-free	1,120 (108)	1,571 (152)	1,441 (140)
R-work	0.240 (0.363)	0.234 (0.401)	0.162 (0.237)
R-free	0.268 (0.360)	0.282 (0.443)	0.183 (0.260)
CC(work)	0.95 (0.86)	0.95 (0.35)	0.97 (0.87)
CC(free)	0.93 (0.84)	0.95 (0.37)	0.97 (0.81)
Number of non-hydrogen atoms	5,032	5,249	7,626
macromolecules	4,729	5,087	6,689
ligands	2	0	39
solvent	301	162	898
Protein residues	628	668	861
RMSD (bonds)	0.017	0.008	0.011
RMSD (angles)	2.02	1.12	1.26
Ramachandran favored (%)	94.63	95.27	98.12
Ramachandran allowed (%)	4.89	4.73	1.88
Ramachandran outliers (%)	0.49	0.00	0.00
Rotamer outliers (%)	2.91	2.32	1.28
Clashscore	5.57	2.67	2.09
Average B-factor	45.72	61.91	23.27
macromolecules	45.65	62.17	21.90
ligands	46.88	n/a	53.03
solvent	46.78	53.74	32.14

^a^Statistics for the highest-resolution shell shown in parentheses.

Although the epitopes bound by the two Fabs were similar, the buried surface areas between the two Fabs and FHbp were different, 916 Å^2^ for the 1A3 complex and 817 Å^2^ for the 7B10 complex. Moreover, there were differences in the polar and charged intermolecular interactions between the two paratopes and their respective epitopes (**S2 Table**). The different interactions corresponded with differences in the amino acid sequences of the CDRs of the heavy and light chains of the two Fabs (**S3 Fig)**. Most notably, there were two salt bridges in the interface of the Fab 7B10-FHbp complex, while there were none in that of the 1A3-FHbp complex. A superposition of the Fab molecules from the two complexes identifies the largest structural differences in CDR-H3 and -L1 (**S4A Fig**). CDR-H3 has backbone shifts of as much as 3 Å between the two complexes. CDR-L1 has a disordered segment in the Fab 7B10 complex that is ordered in the 1A3 complex; there are also backbone differences of up to 5 Å.

To determine whether there were conformational changes associated with Fab binding to FHbp, we crystallized Fab 7B10 alone, and solved its structure to 1.5-Å resolution (**Table 1**). An electron density map in the region of CDR-H3 is shown in **S5 Fig**. A superposition of Fab 7B10 alone with that in its complex with FHbp indicates that the largest rearrangements are in CDR-L1 and -L2 (**S4B Fig**). In the structure of the FHbp complex, CDR-L1 has five disordered residues, which are ordered in the Fab alone. CDR-L2 shows a backbone shift of up to 3 Å between the two structures.

**Fig 4:**
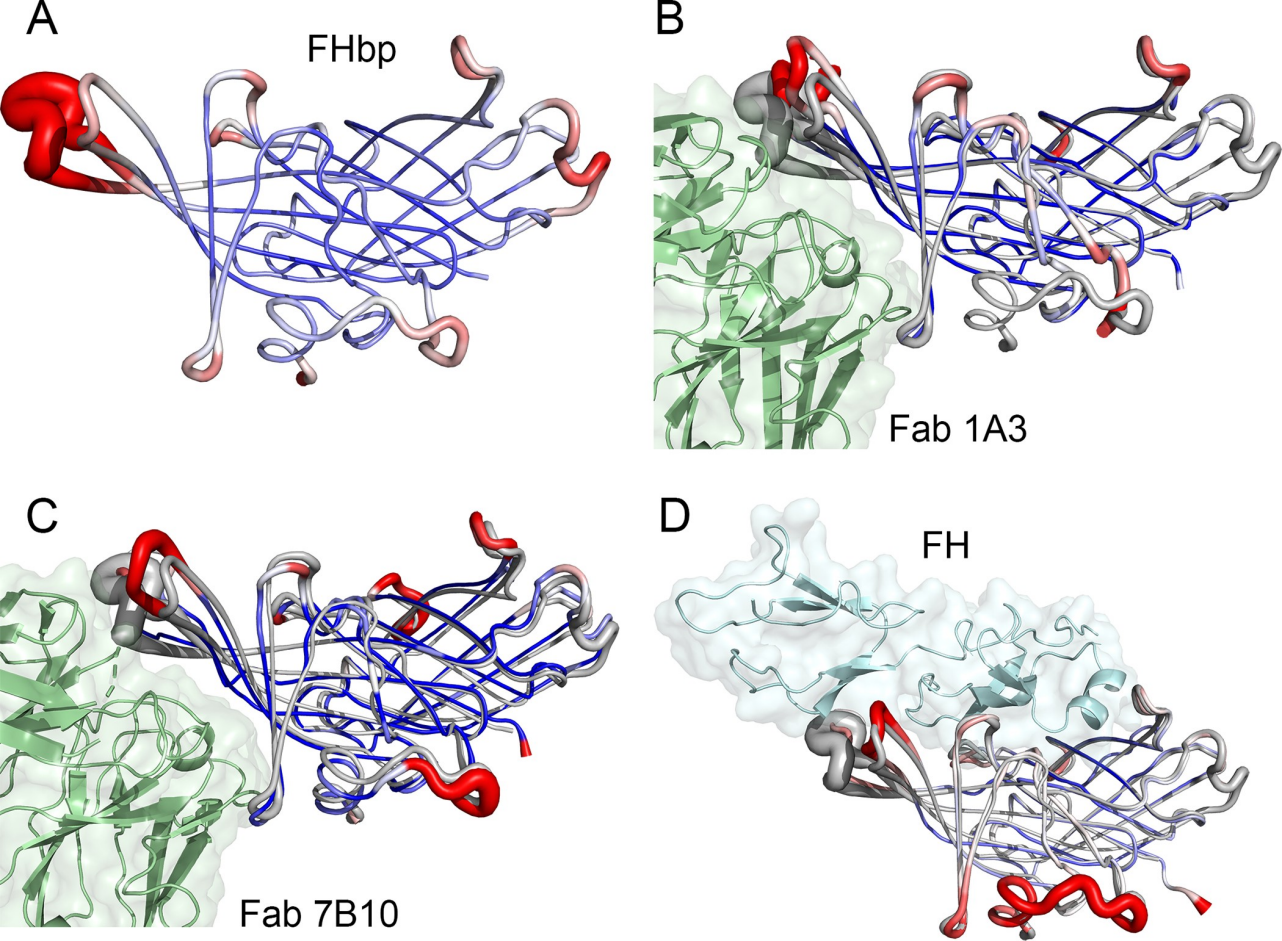
Impact of Fab and FH binding on conformation and thermal (B-) factors of loops involved in intermolecular interactions. (A) FHbp alone (PDB ID 3KVD [51]). (B) FHbp bound to Fab 1A3. (C) FHbp bound to Fab 7B10. (D) FHbp bound to human FH fragment (PDB ID 2W80 [27]). For all panels, high B-factors are shown in red and low B-factors in blue; coloring was scaled based on the mean isotropic B-factor of each structure. For panels B-D, FHbp alone is shown in grey. Figure constructed using PyMol (Schrodinger, LLC).

We also examined the effects of the human Fabs on the conformation and flexibility of loop regions of FHbp using structural superpositions and visualization of crystallographic thermal (B-) factors. Notably, the Fabs affected the conformations and mobilities of two prominent surface-exposed loops compared with the structure of FHbp alone (**Fig 4A–4C**). Each of the human Fabs displaces these two loops of FHbp away from the Fab and decreases the B-factors of backbone atoms in these loops, which is seen as a decrease in the radius of the tube representations (**Fig 4B and 4C**). Interestingly, binding of FH to FHbp also affects the conformations and mobilities of these two loops even though FH binds to an adjacent surface of FHbp compared with the human Fabs (**Fig 4D**).

**Fig 5:**
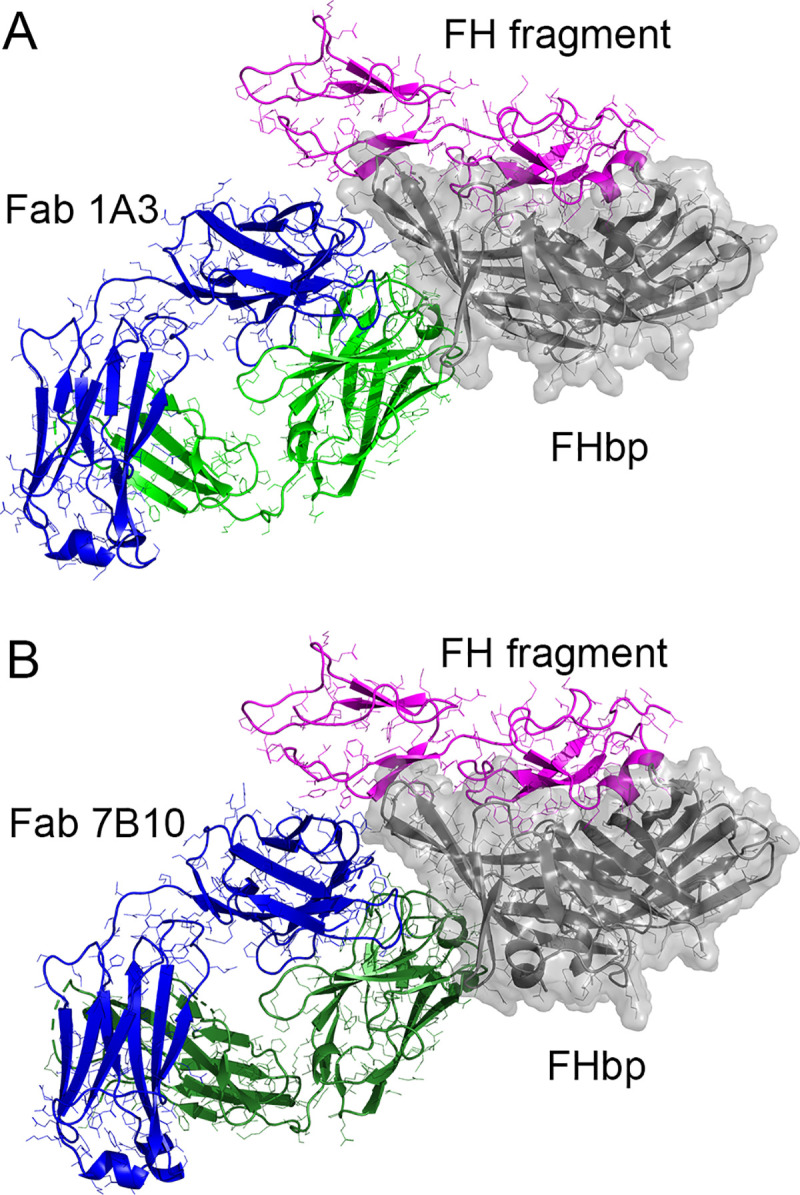
Molecular models showing relative positions of human Fabs and FH. (A) FHbp-Fab 1A3 complex superimposed with FHbp-FH complex PDB ID 2W80 [27]. (B) FHbp-Fab 7B10 complex superimposed with FHbp-FH complex. The coloring scheme and orientation is the same as in Fig 3, and human FH fragment (domains 6 and 7) are shown in magenta. Figure constructed using PyMol (Schrodinger, LLC).

Finally, to evaluate the structural bases for the effects of the Fabs on FH binding to FHbp, we constructed a superposition model using the two Fab-FHbp complexes and that of a fragment of human FH in a complex with FHbp [27] (**Fig 5**). These analyses suggest that the human Fabs are likely to be in direct contact with FH, because the closest polar atoms between Fab and FH in the superposition models are 3.7 and 2.4 Å, for 1A3 and 7B10, respectively. Although these are near and within H-bonding distance, the indirect effects of the human Fabs on the conformation of the FH-binding site might be more important for FH enhancement, which we discuss below.

## Discussion

Meningococcal FHbp is part of two licensed vaccines for prevention of disease caused by serogroup B strains [17,18], and these vaccines also protect against disease caused by several other serogroups of meningococci [28,29]. The serum antibody responses of mice, rabbits, macaques and humans immunized with vaccines containing FHbp have been investigated in depth (e.g. refs. [30–34]). Whereas FHbp elicits serum antibodies in wild-type mice that inhibit FH binding, antibodies from human FH transgenic mice enhance FH binding [19]. Moreover, antibodies from rhesus macaques immunized with FHbp antigens also enhance binding of FH to FHbp [31,35–37]. Similarly, humans immunized with a licensed meningococcal serogroup B vaccine developed antibodies that enhanced FH binding [22]. Whereas most mouse anti-FHbp MAbs inhibit binding of FH to FHbp, the majority of human MAbs to FHbp do not inhibit and in some cases enhance FH binding [38–40]. Therefore, FH enhancing antibodies are produced in all species tested whose FH binds FHbp, including humans.

The crystal structure of an FH fragment comprising domains 6 and 7 bound to FHbp elucidated the FH binding site of FHbp [27]. Moreover, this structure enabled the interactions of mouse and human antibody Fab fragments with FHbp to be interpreted with respect to FH binding or inhibition. Crystal structures of two mouse anti-FHbp Fabs, designated 2C1 and JAR 5, each in a complex with FHbp, showed that the basis for inhibition of FH binding to FHbp was by partially or completely occluding the FH binding site [24,41]. Further, the structures of complexes of two human Fabs, 1A12 and 1E6, with FHbp showed the basis for their broad cross-reactivity with divergent FHbp sequence variants [42,43]. However, neither of these human Fabs inhibited nor enhanced FH binding. A crystal structure of a third human Fab, 4B3, in a complex with FHbp confirmed that the human antibody repertoire does include bactericidal antibodies that inhibit FH binding [44]. This Fab was derived from one of three MAbs that inhibited FH binding out of 110 human anti-FHbp antibodies tested. Thus, in conjunction with our earlier study [22], it appears that the vast majority of human antibodies to FHbp do not inhibit binding of FH, but those that do can elicit bactericidal activity individually [44].

In our previous study, four of ten human anti-FHbp Fabs enhanced FH binding to the bacterial surface by flow cytometry [22]. In the present study, we investigated two of these Fabs more extensively, and we confirmed their ability to enhance FH binding to purified FHbp in two ELISA formats. In all of these experiments, Fab 7B10 gave higher enhancement of FH binding (5- to 10-fold) than did Fab 1A3 (2- to 3-fold). Although we expected that the enhancement of FH binding would result from a higher affinity of FH for the Fab-FHbp complex compared with that of FH for FHbp alone, we observed slightly lower affinity of FH for the complex by SPR. Both of the human Fabs decreased the on-rate (*k*_*a*_) for FH binding and Fab 7B10 also decreased the off-rate (*k*_*d*_). Although we did not measure affinity under equilibrium conditions, it is thought that kinetic SPR experiments yield reliable measurements of affinity [45], particularly at lower coupling densities. One possible explanation for these differences is that the ELISA and flow cytometry experiments measured binding under equilibrium conditions, whereas the SPR experiments measured binding kinetics. Thus, the ELISA and flow cytometry assays might be less sensitive to differences in on-rates but more sensitive to the off-rates, and also because these experiments involve multiple washing steps to remove non-specifically bound antibodies.

Prior to determining the crystal structures of the human Fab complexes, our hypothesis was that the enhancing Fabs stabilized the FH-FHbp complex by direct interactions between Fab and FH. Based on superposition models with the structure of FHbp in a complex with an FH fragment [27], the structures of the Fab-FHbp complexes showed that the Fabs are in proximity to FH and likely are in direct contact. However, judging from the limited contact surface area, the direct interactions are not likely to explain the enhanced FH binding. Rather, it appears that the Fabs lead to structural rearrangements in two loops of FHbp that comprise part of the FH binding site. In addition, based on the crystallographic B-factors, the Fabs also constrain the mobilities of these two loops. The restricted conformational changes potentially explain the lower on- and off-rates for FH binding measured by SPR. More definitive mechanistic details on the enhancement of FH binding could be obtained through structural analyses of ternary complexes of the Fabs with FHbp and FH.

We did not anticipate that the two human Fabs, which differ in many of their properties, would bind to similar epitopes on FHbp. The two Fabs are derived from some of the same germ line immunoglobulin (Ig) genes including heavy and light chain V genes, but not D or J genes (S1 Table in ref. [22]). It is clear that the two Fabs are not clonal based on the fact that they were isolated from different human subjects and they differ in their germline Ig gene usage [22]. In addition, the Fabs contain between two and ten sequence differences in each of the six CDRs (**S3 Fig**). From an analysis of the polar contacts between Fabs and FHbp using PISA [46], 6 of 10 H-bond interactions are the same in both complexes (**S2 Table**). Further, five of the six FHbp residues mediating these polar contacts are conserved among the FHbp phylogenic variant groups, which suggests that the Fabs would bind to most or all FHbp variants. Interestingly, the two salt bridges present in the Fab 7B10 complex but not in the Fab 1A3 complex appear to be compensated for by several additional H-bonds in the latter complex, conferring similar affinities of the two Fabs for FHbp (~1 nM) [22].

Although our study is based on only two FH enhancing Fabs, it raises the question of how many different FH enhancing epitopes exist on the FHbp vaccine antigen. One mouse MAb, MAb 502, is known to enhance binding of FH to FHbp, and the epitope that it recognizes appears to be distinct [47,48] from that bound by the human Fabs described here. Structures of complexes of additional FH enhancing human Fabs would elucidate whether there are any other FH enhancing epitopes. If there were only one or several FH enhancing epitopes, our structural data raise the possibility that the FH enhancing epitopes could be eliminated by mutation of one or two of the FHbp residues that mediate conserved interactions with both Fabs. Collectively, the effects of the two anti-FHbp human Fabs on FH binding kinetics and high-resolution structural data provide new information on an important subset of the human antibody repertoire to FHbp vaccine antigens that might provide insight on strategies to redirect the antibody repertoire away from FH enhancing epitopes. Although we do not yet know the clinical significance of the FH enhancing human Fabs, further studies are needed to determine the net contributions of antibodies that enhance or inhibit binding of FH to meningococcal FHbp.

## Methods

### Production of human and chimeric anti-FHbp Fabs

The human Fabs used in this study have GenBank accession numbers of KP770116, KP770115, KP770118 and KP770117 for the heavy and light chains of 1A3 and 7B10, respectively and were characterized previously [22]. In previous studies, we produced human Fabs in *E*. *coli* [22,42]. For the present studies that required larger quantities of the Fabs, we had two of the human Fabs expressed in mammalian cells (TunaCHO; LakePharma, Inc.). As controls, we used two chimeric Fabs that comprised mouse variable regions (V_H_ and V_L_) derived from mouse MAbs JAR 4 and JAR 5 [23] and human constant regions (C_H_1 and κ_L_), which enabled detection of the human and control Fabs with the same reagents. The Fabs were purified by anti-C_H_1 chromatography and tested for purity by capillary electrophoresis under denaturing conditions, and their concentrations were determined by measuring the UV absorbance at 280 nm and using the molar extinction coefficients calculated from their sequences (https://web.expasy.org/protparam/) [49].

### Enyzme linked immunosorbent assay (ELISA)

Purified, recombinant FHbp (2 μg/ml) was immobilized in the wells of an ELISA microtiter plate (Immulon-2B; ThermoFisher) by incubation at 4°C overnight. The five different FHbp variants used in this study were purified in our laboratory by Ni^2+^-affinity and ion-exchange chromatography as described previously [50]. Non-specific binding was blocked with 5% nonfat dry milk in phosphate-buffered saline (PBS; Roche) containing 0.1% (v/v) Tween-20 (Sigma) for 1 h at room temperature. After washing the plate, serial dilutions of the Fabs or purified human FH were added to the wells and the plate was incubated for 1 h at room temperature. For enhancement experiments, the Fab or FH concentration was fixed at 5 μg/ml (Fig 1B or 1C, respectively). After washing, the bound Fabs were detected with goat anti-human IgG (Fab-specific) conjugated with alkaline phosphatase (AP) (1:10,000; Sigma). Alternatively, bound FH was detected with sheep anti-human FH followed by donkey anti-sheep IgG conjugated with AP (1 h each at room temperature). The colorimetric assay was developed by adding *p*-nitrophenyl phosphate (1 mg/ml; Sigma) in NaCO_2_, 1 mM MgCl_2_, pH 9.8. The ELISAs were performed in three to four independent assays, and the means of triplicate measurements and 2 SE were calculated in Prism 8.3 (GraphPad).

### Surface Plasmon Resonance (SPR)

We performed SPR experiments using FHbp ID 1 (200 to 400 RU) covalently coupled to CM5 biosensor chips and a Biacore X100 Plus instrument (GE Life Sciences). We conducted direct binding experiments using multiple-cycle kinetics and a concentration series of each Fab or purified human FH (Complement Technology, Inc.) from 1 to 100 nM. For experiments to detect enhancement of FH binding by human anti-FHbp Fabs, we injected 10 μM Fab for 120 s to achieve near saturation, then injected a concentration series of 1 to 100 nM of FH. The regeneration solution was 10 mM glycine-HCl, pH 1.7, which was injected for 30 s between protein solutions. The data were analyzed using Biacore Evaluation software with a 1:1 binding model. The data from two to three independent experiments, each performed in duplicate, are reported as means and 2 SE. Probability- (*p*-) values were obtained from two tailed *t*-tests, which were calculated in Prism 8.3 (GraphPad).

### Crystallization, data collection and structure refinement

We formed equimolar complexes of FHbp with the human Fab 1A3, and separately with 7B10, and purified stable complexes by size-exclusion chromatography (HiLoad Superdex 16/60; GE Life Sciences) in 10 mM Tris-HCl, 25 mM NaCl, pH 7.0. We concentrated the Fab-FHbp complexes to 6 to 8 mg/ml by ultrafiltration (Corning Spin-X UF-6, 30K MWCO). We crystallized the complexes by sparse-matrix crystallization screening (Crystal Screen, PEG Ion and Index; Hampton Research Corp.) in sitting-drop crystallization plates (Art Robbins 96-well or MRC 48-well). We optimized the crystallization conditions in hanging drops in 24-well VDX plates. Crystals of the 1A3 complex grew in 0.2 M LiNO_3_, 16% PEG 3,350; crystals of the FHbp-Fab 7B10 complex grew in 0.2 M NaSCN, 20% PEG 3,350; and crystals of Fab 7B10 alone grew in 0.1 M Bis-Tris-Propane pH 5.5, 0.3 M LiSO_4_, 20.5% PEG 3,350.

Crystals of at least 0.02 mm in the smallest dimension were used to collect high-resolution X-ray diffraction data on beamline 8.3.1 at the Advanced Light Source, Lawrence Berkeley National Laboratory. Useful diffraction limits for the 1A3 and 7B10 Fab complexes were 1.7 and 2.0 Å, respectively and for the 7B10 Fab alone was 1.5 Å. Although we were able to obtain small crystals of Fab 1A3 alone, these were not of sufficient quality to obtain useful diffraction data. We solved the three structures by molecular replacement using FHbp ID 1 (3KVD; [51]) and/or homology models based on the sequences of the human Fabs built with Swiss-Model (https://swissmodel.expasy.org) [52]. We solved, refined and validated the structures using the Phenix suite [53] and used Coot [54] for manual model building. Ramachandran and other geometrical outliers were inspected in the electron density maps and were found to be supported by the X-ray data. Structural analyses and generation of Figures were generated with PyMol (The PyMOL Molecular Graphics System, Version 2.4.0, Schrödinger, LLC.). The Protein Data Bank deposition identification numbers, and the data collection and refinement statistics are given in **Table 1**.

## Supporting information

S1 Table Kinetic effects of human Fabs on FH binding to FHbp. Means and SE of 4 to 6 replicates are shown.
(DOCX)

S2 Table Charged or polar atomic interactions between human Fabs and FHbp. Calculated with Proteins, Interfaces, Structures and Assemblies (PISA) server (https://www.ebi.ac.uk/pdbe/pisa/) [46]. Salt bridges are shown in bold and H-bonds are shown in regular type. Chain names from PDB coordinate files: A, Fab heavy chain; B, Fab light chain; C, FHbp. FHbp numbering is based on the amino acid sequence of the mature lipoprotein, beginning with the lipidated Cys residue.
(DOCX)

S1 Fig Cross-reactivity of binding of human anti-FHbp Fabs to diverse FHbp sequence variants by ELISA. Human or chimeric control Fabs were tested for binding with FHbp sequence variants ID 1, 13, 14, 22, and 55. (A) Chimeric human-mouse Fab JAR 5. (B) Chimeric human-mouse Fab JAR 4. (C) Human Fab 1A3. (D) Human Fab 7B10. Bound Fab was detected with anti-human IgG (Fab-specific) antibody conjugated to alkaline phosphatase (Sigma 1:5,000). The means and 2SE of triplicate measurements are shown.
(TIF)

S2 Fig Binding of FH to FHbp in the absence or presence of human anti-FHbp Fabs. (A) Binding of FH in the absence of human anti-FHbp Fab. (B) Binding of FH in the presence of human anti-FHbp Fab 7B10. (C) Binding of FH in the presence of human anti-FHbp Fab 1A3. Duplicate runs from one of at least four independent experiments are shown.
(TIF)

S3 Fig Amino acid sequence alignment of human Fabs. (A) Heavy chain Fd fragment. (B) kappa light chains. The complementarity determining regions (CDR) of the heavy (H) and light (L) chains are shown in bold type. Accession numbers of the Fab sequences are given in Methods and alignment was performed with Clustal Omega [55].
(PDF)

S4 Fig Atomic interactions between Fab 1A3 and FHbp. The bonds in the CDR loops of the heavy chain (chain A) are shown in different shades of green; bonds in the CDR-L1 loop of the light chain (B) are shown in aqua; bonds of the Fab outside of the CDR loops are shown in dark slate; bonds of FHbp are shown in magenta; and water molecules are shown in light blue. The Figure was generated using the Antibody feature in LigPlot+ and CDR loops were defined by Kabat convention implemented in LigPlot+ [56].
(PDF)

S5 Fig Atomic interactions between Fab 7B10 and FHbp. The color scheme is the same as in S4 Fig. The Figure was generated using the Antibody feature in LigPlot+ and CDR loops were defined by Kabat convention implemented in LigPlot+ [56].
(PDF)

S6 Fig Structural comparisons of human Fabs. (A) FHbp-Fab 7B10 complex superimposed with FHbp-Fab 1A3 complex. (B) FHbp-Fab 7B10 complex superimposed with Fab 7B10 alone. For clarity, in both panels only the Fab molecules are shown. Figure generated with PyMol (Schrodinger, LLC).
(TIF)

S7 Fig Electron density map of human Fab 7B10 alone in the region of CDR-H3. The 2Fo-Fc feature enhanced map [57] was calculated in Phenix [53] and the map is contoured at 1.2 sigma. Figure generated with PyMol (Schrodinger, LLC).
(TIF)

## References

[1] Schneider MC, Exley RM, Chan H, Feavers I, Kang YH, Sim RB, et al. Functional significance of Factor H binding to *Neisseria meningitidis*. J Immunol. 2006;176(12):7566–75. Epub 2006/06/06. doi: 10.4049/jimmunol.176.12.7566 . 16751403

[2] Meri S, Jordens M, Jarva H. Microbial complement inhibitors as vaccines. Vaccine. 2008;26 Suppl 8:I113–7. Epub 2009/04/24. doi: 10.1016/j.vaccine.2008.11.058 . 19388175

[3] Makou E, Herbert AP, Barlow PN. Functional anatomy of complement factor H. Biochemistry. 2013;52(23):3949–62. Epub 2013/05/25. doi: 10.1021/bi4003452 . 23701234

[4] Wong EKS, Hallam TM, Brocklebank V, Walsh PR, Smith-Jackson K, Shuttleworth VG, et al. Functional characterization of rare genetic variants in the N-terminus of complement Factor H in aHUS, C3G, and AMD. Front Immunol. 2020;11:602284. Epub 2021/02/02. doi: 10.3389/fimmu.2020.602284 ; PubMed Central PMCID: PMC7840601. 33519811 PMC7840601

[5] Kerr H, Wong E, Makou E, Yang Y, Marchbank K, Kavanagh D, et al. Disease-linked mutations in factor H reveal pivotal role of cofactor activity in self-surface-selective regulation of complement activation. J Biol Chem. 2017;292(32):13345–60. Epub 2017/06/24. doi: 10.1074/jbc.M117.795088 ; PubMed Central PMCID: PMC5555194. 28637873 PMC5555194

[6] Dopler A, Guntau L, Harder MJ, Palmer A, Hochsmann B, Schrezenmeier H, et al. Self versus nonself discrimination by the soluble complement regulators Factor H and FHL-1. J Immunol. 2019;202(7):2082–94. Epub 2019/02/13. doi: 10.4049/jimmunol.1801545 . 30745459

[7] Parente R, Clark SJ, Inforzato A, Day AJ. Complement factor H in host defense and immune evasion. Cell Mol Life Sci. 2017;74(9):1605–24. Epub 2016/12/13. doi: 10.1007/s00018-016-2418-4 ; PubMed Central PMCID: PMC5378756. 27942748 PMC5378756

[8] Madico G, Welsch JA, Lewis LA, McNaughton A, Perlman DH, Costello CE, et al. The meningococcal vaccine candidate GNA1870 binds the complement regulatory protein factor H and enhances serum resistance. J Immunol. 2006;177(1):501–10. Epub 2006/06/21. doi: 10.4049/jimmunol.177.1.501 ; PubMed Central PMCID: PMC2248442. 16785547 PMC2248442

[9] Lewis LA, Ngampasutadol J, Wallace R, Reid JE, Vogel U, Ram S. The meningococcal vaccine candidate Neisserial surface protein A (NspA) binds to factor H and enhances meningococcal resistance to complement. PLoS Pathog. 2010;6(7):e1001027. Epub 2010/08/06. doi: 10.1371/journal.ppat.1001027 ; PubMed Central PMCID: PMC2912398. 20686663 PMC2912398

[10] Lewis LA, Vu DM, Granoff DM, Ram S. Inhibition of the alternative pathway of nonhuman infant complement by porin B2 contributes to virulence of Neisseria meningitidis in the infant rat model. Infect Immun. 2014;82(6):2574–84. Epub 2014/04/02. doi: 10.1128/IAI.01517-14 ; PubMed Central PMCID: PMC4019150. 24686052 PMC4019150

[11] Masignani V, Comanducci M, Giuliani MM, Bambini S, Adu-Bobie J, Arico B, et al. Vaccination against *Neisseria meningitidis* using three variants of the lipoprotein GNA1870. J Exp Med. 2003;197(6):789–99. Epub 2003/03/19. doi: 10.1084/jem.20021911 ; PubMed Central PMCID: PMC2193853. 12642606 PMC2193853

[12] Fletcher LD, Bernfield L, Barniak V, Farley JE, Howell A, Knauf M, et al. Vaccine potential of the *Neisseria meningitidis* 2086 lipoprotein. Infect Immun. 2004;72(4):2088–100. Epub 2004/03/25. doi: 10.1128/IAI.72.4.2088-2100.2004 ; PubMed Central PMCID: PMC375149. 15039331 PMC375149

[13] Lewis LA, Carter M, Ram S. The relative roles of factor H binding protein, Neisserial surface protein A, and lipooligosaccharide sialylation in regulation of the alternative pathway of complement on meningococci. J Immunol. 2012;188(10):5063–72. Epub 2012/04/17. doi: 10.4049/jimmunol.1103748 ; PubMed Central PMCID: PMC3345070. 22504643 PMC3345070

[14] Granoff DM, Welsch JA, Ram S. Binding of complement factor H (fH) to *Neisseria meningitidis* is specific for human fH and inhibits complement activation by rat and rabbit sera. Infect Immun. 2009;77(2):764–9. Epub 2008/12/03. doi: 10.1128/IAI.01191-08 ; PubMed Central PMCID: PMC2632036. 19047406 PMC2632036

[15] Beernink PT, Shaughnessy J, Stefek H, Ram S, Granoff DM. Heterogeneity in rhesus macaque complement factor H binding to meningococcal factor H binding protein (FHbp) informs selection of primates to assess immunogenicity of FHbp-based vaccines. Clin Vaccine Immunol. 2014;21(11):1505–11. Epub 2014/09/05. doi: 10.1128/CVI.00517-14 ; PubMed Central PMCID: PMC4248754. 25185576 PMC4248754

[16] Beernink PT, Shaughnessy J, Braga EM, Liu Q, Rice PA, Ram S, et al. A meningococcal factor H binding protein mutant that eliminates factor H binding enhances protective antibody responses to vaccination. J Immunol. 2011;186(6):3606–14. Epub 2011/02/18. doi: 10.4049/jimmunol.1003470 ; PubMed Central PMCID: PMC3098282. 21325619 PMC3098282

[17] Granoff DM. Commentary: European Medicines Agency recommends approval of a broadly protective vaccine against serogroup B meningococcal disease. Pediatr Infect Dis J. 2013;32(4):372–3. Epub 2012/12/25. doi: 10.1097/INF.0b013e318282942f . 23263177

[18] Folaranmi T, Rubin L, Martin SW, Patel M, MacNeil JR, Centers for Disease C. Use of serogroup B meningococcal vaccines in persons aged >/ = 10 years at increased risk for serogroup B meningococcal disease: Recommendations of the Advisory Committee on Immunization Practices, 2015. MMWR Morb Mortal Wkly Rep. 2015;64(22):608–12. Epub 2015/06/13. ; PubMed Central PMCID: PMC4584923. 26068564 PMC4584923

[19] Costa I, Pajon R, Granoff DM. Human factor H (FH) impairs protective meningococcal anti-FHbp antibody responses and the antibodies enhance FH binding. mBio. 2014;5(5):e01625–14. Epub 2014/08/28. doi: 10.1128/mBio.01625-14 ; PubMed Central PMCID: PMC4173785. 25161192 PMC4173785

[20] Lujan E, Pajon R, Granoff DM. Impaired immunogenicity of meningococcal Neisserial surface protein A in human complement Factor H transgenic mice. Infect Immun. 2016;84(2):452–8. Epub 2015/11/26. doi: 10.1128/IAI.01267-15 ; PubMed Central PMCID: PMC4730568. 26597984 PMC4730568

[21] van der Veen S, Johnson S, Jongerius I, Malik T, Genovese A, Santini L, et al. Nonfunctional variant 3 factor H binding proteins as meningococcal vaccine candidates. Infect Immun. 2014;82(3):1157–63. Epub 2014/01/01. doi: 10.1128/IAI.01183-13 ; PubMed Central PMCID: PMC3958001. 24379280 PMC3958001

[22] Beernink PT, Giuntini S, Costa I, Lucas AH, Granoff DM. Functional analysis of the human antibody response to meningococcal Factor H binding protein. mBio. 2015;6(3):e00842. Epub 2015/06/25. doi: 10.1128/mBio.00842-15 ; PubMed Central PMCID: PMC4479705. 26106082 PMC4479705

[23] Welsch JA, Rossi R, Comanducci M, Granoff DM. Protective activity of monoclonal antibodies to genome-derived Neisserial antigen 1870, a *Neisseria meningitidis* candidate vaccine. J Immunol. 2004;172(9):5606–15. Epub 2004/04/22. doi: 10.4049/jimmunol.172.9.5606 . 15100304

[24] Malito E, Lo Surdo P, Veggi D, Santini L, Stefek H, Brunelli B, et al. *Neisseria meningitidis* factor H-binding protein bound to monoclonal antibody JAR5: implications for antibody synergy. Biochem J. 2016;473(24):4699–713. Epub 2016/10/28. doi: 10.1042/BCJ20160806 ; PubMed Central PMCID: PMC6398935. 27784765 PMC6398935

[25] Beernink PT, LoPasso C, Angiolillo A, Felici F, Granoff D. A region of the N-terminal domain of meningococcal factor H-binding protein that elicits bactericidal antibody across antigenic variant groups. Mol Immunol. 2009;46(8–9):1647–53. Epub 2009/03/17. doi: 10.1016/j.molimm.2009.02.021 ; PubMed Central PMCID: PMC2673115. 19286260 PMC2673115

[26] Beernink PT, Granoff DM. The modular architecture of meningococcal factor H-binding protein. Microbiology (Reading). 2009;155(Pt 9):2873–83. Epub 2009/07/04. doi: 10.1099/mic.0.029876-0 ; PubMed Central PMCID: PMC2859308. 19574307 PMC2859308

[27] Schneider MC, Prosser BE, Caesar JJ, Kugelberg E, Li S, Zhang Q, et al. *Neisseria meningitidis* recruits factor H using protein mimicry of host carbohydrates. Nature. 2009;458(7240):890–3. Epub 2009/02/20. doi: 10.1038/nature07769 ; PubMed Central PMCID: PMC2670278. 19225461 PMC2670278

[28] Harris SL, Tan C, Andrew L, Hao L, Liberator PA, Absalon J, et al. The bivalent factor H binding protein meningococcal serogroup B vaccine elicits bactericidal antibodies against representative non-serogroup B meningococci. Vaccine. 2018;36(45):6867–74. Epub 2018/10/03. doi: 10.1016/j.vaccine.2018.05.081 . 30269916

[29] Biolchi A, Tomei S, Brunelli B, Giuliani M, Bambini S, Borrow R, et al. 4CMenB immunization induces serum bactericidal antibodies against non-serogroup B meningococcal strains in adolescents. Infect Dis Ther. 2020. Epub 2020/11/14. doi: 10.1007/s40121-020-00370-x . 33185849 PMC7954916

[30] Konar M, Granoff DM, Beernink PT. Importance of inhibition of binding of complement factor H for serum bactericidal antibody responses to meningococcal factor H-binding protein vaccines. J Infect Dis. 2013;208(4):627–36. Epub 2013/05/30. doi: 10.1093/infdis/jit239 ; PubMed Central PMCID: PMC3719908. 23715659 PMC3719908

[31] Granoff DM, Giuntini S, Gowans FA, Lujan E, Sharkey K, Beernink PT. Enhanced protective antibody to a mutant meningococcal factor H-binding protein with low-factor H binding. JCI Insight. 2016;1(14):e88907. Epub 2016/09/27. doi: 10.1172/jci.insight.88907 ; PubMed Central PMCID: PMC5033880. 27668287 PMC5033880

[32] Jiang HQ, Hoiseth SK, Harris SL, McNeil LK, Zhu D, Tan C, et al. Broad vaccine coverage predicted for a bivalent recombinant factor H binding protein based vaccine to prevent serogroup B meningococcal disease. Vaccine. 2010;28(37):6086–93. Epub 2010/07/14. doi: 10.1016/j.vaccine.2010.06.083 . 20619376

[33] Brunelli B, Del Tordello E, Palumbo E, Biolchi A, Bambini S, Comanducci M, et al. Influence of sequence variability on bactericidal activity sera induced by Factor H binding protein variant 1.1. Vaccine. 2011;29(5):1072–81. Epub 2010/12/07. doi: 10.1016/j.vaccine.2010.11.064 . 21130753

[34] Seib KL, Brunelli B, Brogioni B, Palumbo E, Bambini S, Muzzi A, et al. Characterization of diverse subvariants of the meningococcal factor H (fH) binding protein for their ability to bind fH, to mediate serum resistance, and to induce bactericidal antibodies. Infect Immun. 2011;79(2):970–81. Epub 2010/12/15. doi: 10.1128/IAI.00891-10 ; PubMed Central PMCID: PMC3028832. 21149595 PMC3028832

[35] Granoff DM, Costa I, Konar M, Giuntini S, Van Rompay KK, Beernink PT. Binding of complement Factor H (FH) decreases protective anti-FH binding protein antibody responses of infant rhesus macaques immunized with a meningococcal serogroup B vaccine. J Infect Dis. 2015;212(5):784–92. Epub 2015/02/14. doi: 10.1093/infdis/jiv081 ; PubMed Central PMCID: PMC4539902. 25676468 PMC4539902

[36] Giuntini S, Beernink PT, Granoff DM. Effect of complement Factor H on anti-FHbp serum bactericidal antibody responses of infant rhesus macaques boosted with a licensed meningococcal serogroup B vaccine. Vaccine. 2015;33(51):7168–75. Epub 2015/11/13. doi: 10.1016/j.vaccine.2015.10.135 ; PubMed Central PMCID: PMC4684420. 26562320 PMC4684420

[37] Beernink PT, Vianzon V, Lewis LA, Moe GR, Granoff DM. A meningococcal outer membrane vesicle vaccine with overexpressed mutant FHbp elicits higher protective antibody responses in infant rhesus macaques than a licensed serogroup B vaccine. mBio. 2019;10(3). Epub 2019/06/20. doi: 10.1128/mBio.01231-19 ; PubMed Central PMCID: PMC6581866. 31213564 PMC6581866

[38] Beernink PT, Welsch JA, Bar-Lev M, Koeberling O, Comanducci M, Granoff DM. Fine antigenic specificity and cooperative bactericidal activity of monoclonal antibodies directed at the meningococcal vaccine candidate factor h-binding protein. Infect Immun. 2008;76(9):4232–40. Epub 2008/07/02. doi: 10.1128/IAI.00367-08 ; PubMed Central PMCID: PMC2519416. 18591239 PMC2519416

[39] Vu DM, Pajon R, Reason DC, Granoff DM. A broadly cross-reactive monoclonal antibody against an epitope on the N-terminus of meningococcal fHbp. Sci Rep. 2012;2:341. Epub 2012/03/31. doi: 10.1038/srep00341 ; PubMed Central PMCID: PMC3314305. 22461972 PMC3314305

[40] Giuliani M, Bartolini E, Galli B, Santini L, Lo Surdo P, Buricchi F, et al. Human protective response induced by meningococcus B vaccine is mediated by the synergy of multiple bactericidal epitopes. Sci Rep. 2018;8(1):3700. Epub 2018/03/01. doi: 10.1038/s41598-018-22057-7 ; PubMed Central PMCID: PMC5829249. 29487324 PMC5829249

[41] Malito E, Faleri A, Lo Surdo P, Veggi D, Maruggi G, Grassi E, et al. Defining a protective epitope on factor H binding protein, a key meningococcal virulence factor and vaccine antigen. Proc Natl Acad Sci U S A. 2013;110(9):3304–9. Epub 2013/02/12. doi: 10.1073/pnas.1222845110 ; PubMed Central PMCID: PMC3587270. 23396847 PMC3587270

[42] Lopez-Sagaseta J, Beernink PT, Bianchi F, Santini L, Frigimelica E, Lucas AH, et al. Crystal structure reveals vaccine elicited bactericidal human antibody targeting a conserved epitope on meningococcal fHbp. Nat Commun. 2018;9(1):528. Epub 2018/02/08. doi: 10.1038/s41467-018-02827-7 ; PubMed Central PMCID: PMC5802752. 29410413 PMC5802752

[43] Bianchi F, Veggi D, Santini L, Buricchi F, Bartolini E, Lo Surdo P, et al. Cocrystal structure of meningococcal factor H binding protein variant 3 reveals a new crossprotective epitope recognized by human mAb 1E6. FASEB J. 2019;33(11):12099–111. Epub 2019/08/24. doi: 10.1096/fj.201900374R ; PubMed Central PMCID: PMC6902690. 31442074 PMC6902690

[44] Veggi D, Bianchi F, Santini L, Lo Surdo P, Chesterman CC, Pansegrau W, et al. 4CMenB vaccine induces elite cross-protective human antibodies that compete with human factor H for binding to meningococcal fHbp. PLoS Pathog. 2020;16(10):e1008882. Epub 2020/10/03. doi: 10.1371/journal.ppat.1008882 . 33007046 PMC7556464

[45] Day YS, Baird CL, Rich RL, Myszka DG. Direct comparison of binding equilibrium, thermodynamic, and rate constants determined by surface- and solution-based biophysical methods. Protein Sci. 2002;11(5):1017–25. Epub 2002/04/23. doi: 10.1110/ps.4330102 ; PubMed Central PMCID: PMC2373566. 11967359 PMC2373566

[46] Krissinel E, Henrick K. Inference of macromolecular assemblies from crystalline state. J Mol Biol. 2007;372(3):774–97. Epub 2007/08/08. doi: 10.1016/j.jmb.2007.05.022 . 17681537

[47] Scarselli M, Cantini F, Santini L, Veggi D, Dragonetti S, Donati C, et al. Epitope mapping of a bactericidal monoclonal antibody against the factor H binding protein of *Neisseria meningitidis*. J Mol Biol. 2009;386(1):97–108. Epub 2008/12/23. doi: 10.1016/j.jmb.2008.12.005 . 19100746

[48] Giuntini S, Beernink PT, Reason DC, Granoff DM. Monoclonal antibodies to meningococcal factor H binding protein with overlapping epitopes and discordant functional activity. PLoS One. 2012;7(3):e34272. Epub 2012/03/31. doi: 10.1371/journal.pone.0034272 ; PubMed Central PMCID: PMC3312907. 22461909 PMC3312907

[49] Gasteiger E, Hoogland C, Gattiker A, Duvaud S, Wilkins MR, Appel RD, et al. Protein Identification and Analysis Tools on the ExPASy Server. The Proteomics Protocols Handbook: Humana Press; 2005. p. 571–607.

[50] Konar M, Pajon R, Beernink PT. A meningococcal vaccine antigen engineered to increase thermal stability and stabilize protective epitopes. Proc Natl Acad Sci U S A. 2015;112(48):14823–8. Epub 2015/12/03. doi: 10.1073/pnas.1507829112 ; PubMed Central PMCID: PMC4672778. 26627237 PMC4672778

[51] Cendron L, Veggi D, Girardi E, Zanotti G. Structure of the uncomplexed Neisseria meningitidis factor H-binding protein fHbp (rLP2086). Acta Crystallogr Sect F Struct Biol Cryst Commun. 2011;67(Pt 5):531–5. Epub 2011/05/06. doi: 10.1107/S1744309111006154 ; PubMed Central PMCID: PMC3087634. 21543855 PMC3087634

[52] Waterhouse A, Bertoni M, Bienert S, Studer G, Tauriello G, Gumienny R, et al. SWISS-MODEL: homology modelling of protein structures and complexes. Nucleic Acids Res. 2018;46(W1):W296–W303. Epub 2018/05/23. doi: 10.1093/nar/gky427 ; PubMed Central PMCID: PMC6030848. 29788355 PMC6030848

[53] Liebschner D, Afonine PV, Baker ML, Bunkoczi G, Chen VB, Croll TI, et al. Macromolecular structure determination using X-rays, neutrons and electrons: recent developments in Phenix. Acta Crystallogr D Struct Biol. 2019;75(Pt 10):861–77. Epub 2019/10/08. doi: 10.1107/S2059798319011471 ; PubMed Central PMCID: PMC6778852. 31588918 PMC6778852

[54] Emsley P, Lohkamp B, Scott WG, Cowtan K. Features and development of Coot. Acta Crystallogr D Biol Crystallogr. 2010;66(Pt 4):486–501. Epub 2010/04/13. doi: 10.1107/S0907444910007493 ; PubMed Central PMCID: PMC2852313. 20383002 PMC2852313

[55] Sievers F, Wilm A, Dineen D, Gibson TJ, Karplus K, Li W, et al. Fast, scalable generation of high-quality protein multiple sequence alignments using Clustal Omega. Mol Syst Biol. 2011;7:539. Epub 2011/10/13. doi: 10.1038/msb.2011.75 ; PubMed Central PMCID: PMC3261699. 21988835 PMC3261699

[56] Wallace AC, Laskowski RA, Thornton JM. LIGPLOT: a program to generate schematic diagrams of protein-ligand interactions. Protein Eng. 1995;8(2):127–34. Epub 1995/02/01. doi: 10.1093/protein/8.2.127 . 7630882

[57] Afonine PV, Moriarty NW, Mustyakimov M, Sobolev OV, Terwilliger TC, Turk D, et al. FEM: feature-enhanced map. Acta Crystallogr D Biol Crystallogr. 2015;71(Pt 3):646–66. Epub 2015/03/12. doi: 10.1107/S1399004714028132 ; PubMed Central PMCID: PMC4356370. 25760612 PMC4356370

