# Supplementary material for: Two human antibodies to a meningococcal serogroup B vaccine antigen enhance binding of complement Factor H by stabilizing the Factor H binding site
Source: PLoS Pathog. 2021 Jun 14;17(6):e1009655. doi: 10.1371/journal.ppat.1009655 (PMC8224966; doi:10.1371/journal.ppat.1009655)
Supplement: S1 Table — Means and SE of 4 to 6 replicates are shown. (DOCX) [file ppat.1009655.s001.docx]

**S1 Table.** Kinetic effects of human Fabs on FH binding to FHbp^a^

| **Fab** | ***k_a_* x10^-5^ (M^-1^s^-1^)** | ***k_d_* x10^3^ (s^-1^)** | ***K_D_* x10^9^ (M)** | ***R_max_* (RU)** | ***Chi^2^* (RU^2^)** |
| --- | --- | --- | --- | --- | --- |
| None | 7.62 ±0.67 | 2.86 ±0.05 | 3.91 ±0.37 | 36.1 ±0.6 | 3.2 ±0.6 |
| 1A3 | 5.39 ±0.89 | 3.22 ±0.06 | 66.0 ±13.4 | 31.1 ±3.9 | 0.07 ±0.01 |
| 7B10 | 20.1 ±0.1 | 1.45 ±0.01 | 7.26 ±0.30 | 50.7 ±1.0 | 1.9 ±0.3 |

^a^ Means and SE of 4 to 6 replicates are shown
