# Supplementary material for: Two human antibodies to a meningococcal serogroup B vaccine antigen enhance binding of complement Factor H by stabilizing the Factor H binding site
Source: PLoS Pathog. 2021 Jun 14;17(6):e1009655. doi: 10.1371/journal.ppat.1009655 (PMC8224966; doi:10.1371/journal.ppat.1009655)
Supplement: S2 Table — Calculated with Proteins, Interfaces, Structures and Assemblies (PISA) server (https://www.ebi.ac.uk/pdbe/pisa/) [46]. Salt bridges are shown in bold and H-bonds are shown in regular type. Chain names from PDB coordinate files: A, Fab heavy chain; B, Fab light chain; C, FHbp. FHbp numbering is based on the amino acid sequence of the mature lipoprotein, beginning with the lipidated Cys residue. (DOCX) [file ppat.1009655.s002.docx]

**S2 Table.** Charged or polar atomic interactions between human Fabs and FHbp^a^

| **FHbp atom** | **Fab 7B10 atom^b^** |  | **FHbp atom^a^** | **Fab 1A3 atom^b^** |
| --- | --- | --- | --- | --- |
| C:Gln 51 [H] | A:Gly 56: [O] |  | C:Gln 51 [HE22] | A:Gly 56 [O] |
| C:Gln 51 [O] | A:Thr 58 [H] |  | C:Gln 51 [O] | A:Ser 58 [H] |
| **C:Glu 54 [OE1]** | **A:Lys 59 [NZ]** |  | C:Glu 54 [OE2] | A:Tyr 103 [HH] |
| C:Glu 54 [OE1] | A: Lys 59 [HZ1] |  | C:Glu 54 [OE1] | A:Tyr 105 [HH] |
| C:Glu 54 [OE1] | A:Tyr 103 [HH] |  | C:Glu 112 [OE1] | A:Ser 31 [OG] |
| **C:Asp 77 [OD1]** | **A:His 53 [ND1]** |  | C:Gln 113 [HE22] | A:Ser 31 [O] |
| C:Glu 112 [OE1] | A:Ser 31 [OG] |  | C:Gln 113 [OE1] | A:Ser 102 [H] |
| C:Gln 113 [HE22] | A:Ser 31 [O] |  | C:Gln 113 [OE1] | A:Ser 102 [OG] |
| C:Gln 113 [OE1] | A:Ser 102 [H] |  | C:Val 243 [O] | B:Gln 157 [HE22] |
| C:Ser 120 [O] | B:Asn 55 [HD21] |  | C:Asn 244 [OD1] | B:His 191 [NE2] |

^a^ Calculated with Proteins, Interfaces, Structures and Assemblies (PISA) server (<https://www.ebi.ac.uk/pdbe/pisa/>)[45]. Salt bridges are shown in bold and H-bonds are shown in regular type. Chain names from PDB coordinate files: A, Fab heavy chain; B, Fab light chain; C, FHbp. FHbp numbering is based on the amino acid sequence of the mature lipoprotein, beginning with the lipidated Cys residue.
