# Supplementary figures and images for: Two human antibodies to a meningococcal serogroup B vaccine antigen enhance binding of complement Factor H by stabilizing the Factor H binding site
Source: PLoS Pathog. 2021 Jun 14;17(6):e1009655. doi: 10.1371/journal.ppat.1009655 (PMC8224966; doi:10.1371/journal.ppat.1009655)

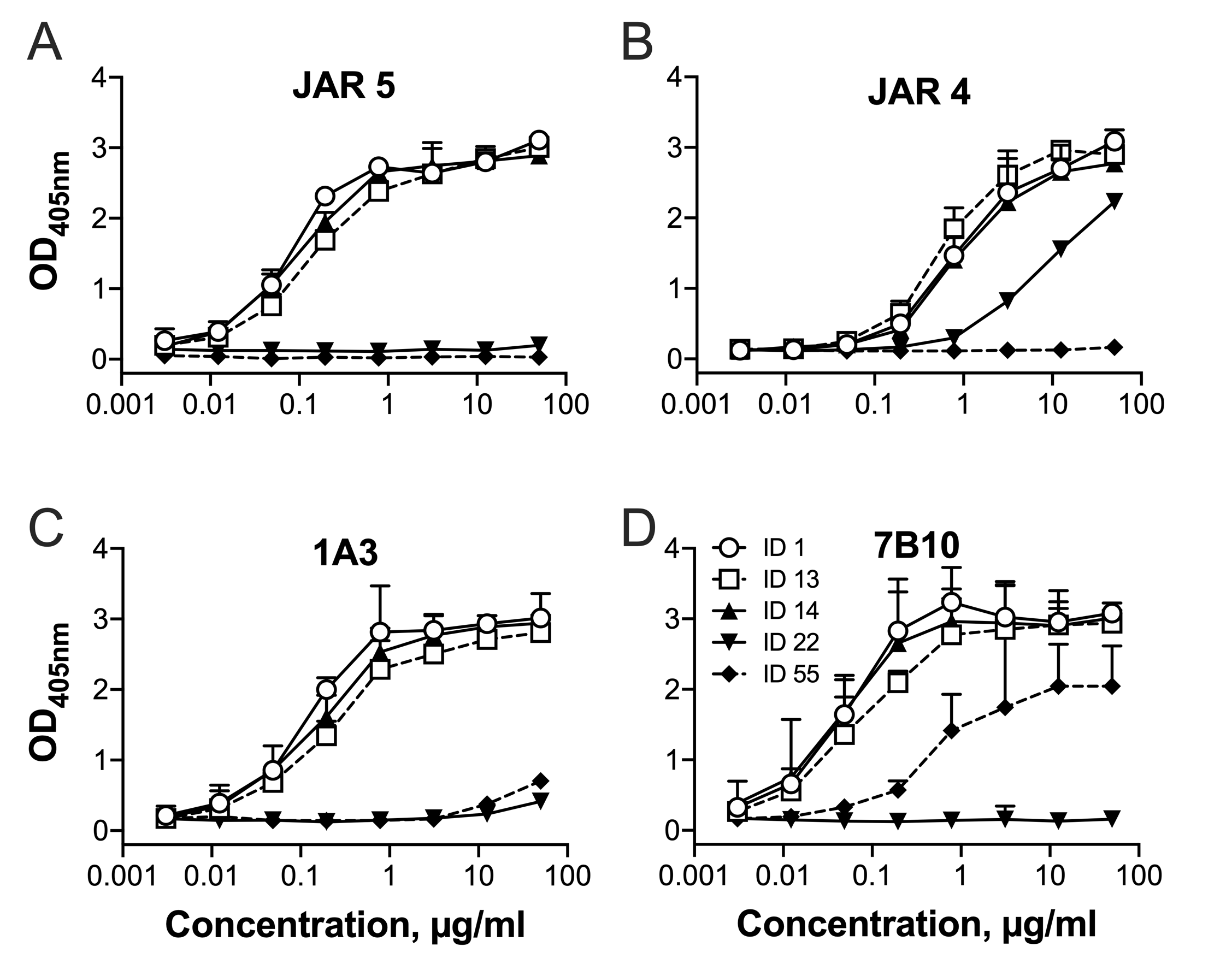

Supplement: S1 Fig — Human or chimeric control Fabs were tested for binding with FHbp sequence variants ID 1, 13, 14, 22, and 55. (A) Chimeric human-mouse Fab JAR 5. (B) Chimeric human-mouse Fab JAR 4. (C) Human Fab 1A3. (D) Human Fab 7B10. Bound Fab was detected with anti-human IgG (Fab-specific) antibody conjugated to alkaline phosphatase (Sigma 1:5,000). The means and 2SE of triplicate measurements are shown. (TIF) [file ppat.1009655.s003.tif]

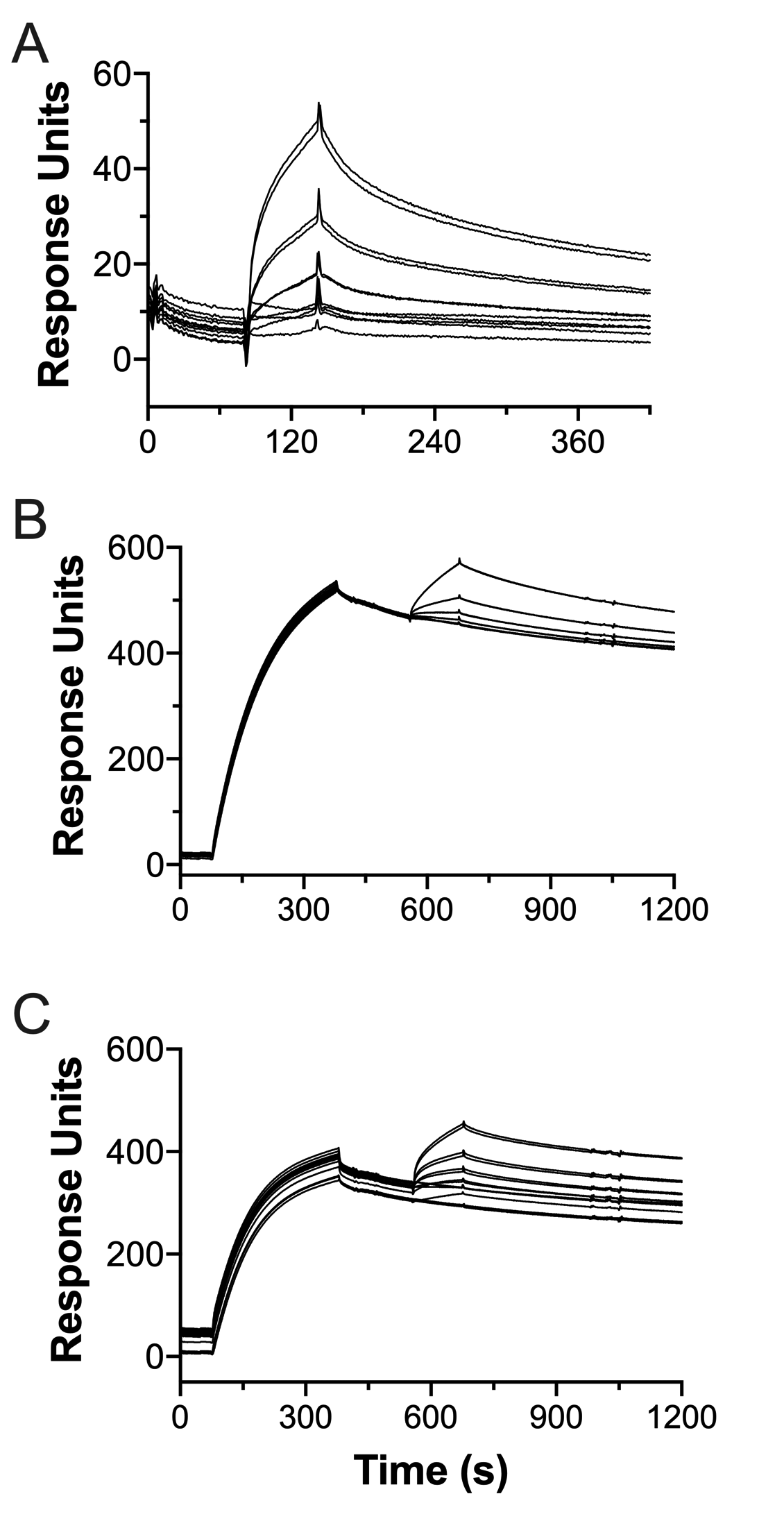

Supplement: S2 Fig — (A) Binding of FH in the absence of human anti-FHbp Fab. (B) Binding of FH in the presence of human anti-FHbp Fab 7B10. (C) Binding of FH in the presence of human anti-FHbp Fab 1A3. Duplicate runs from one of at least four independent experiments are shown. (TIF) [file ppat.1009655.s004.tif]

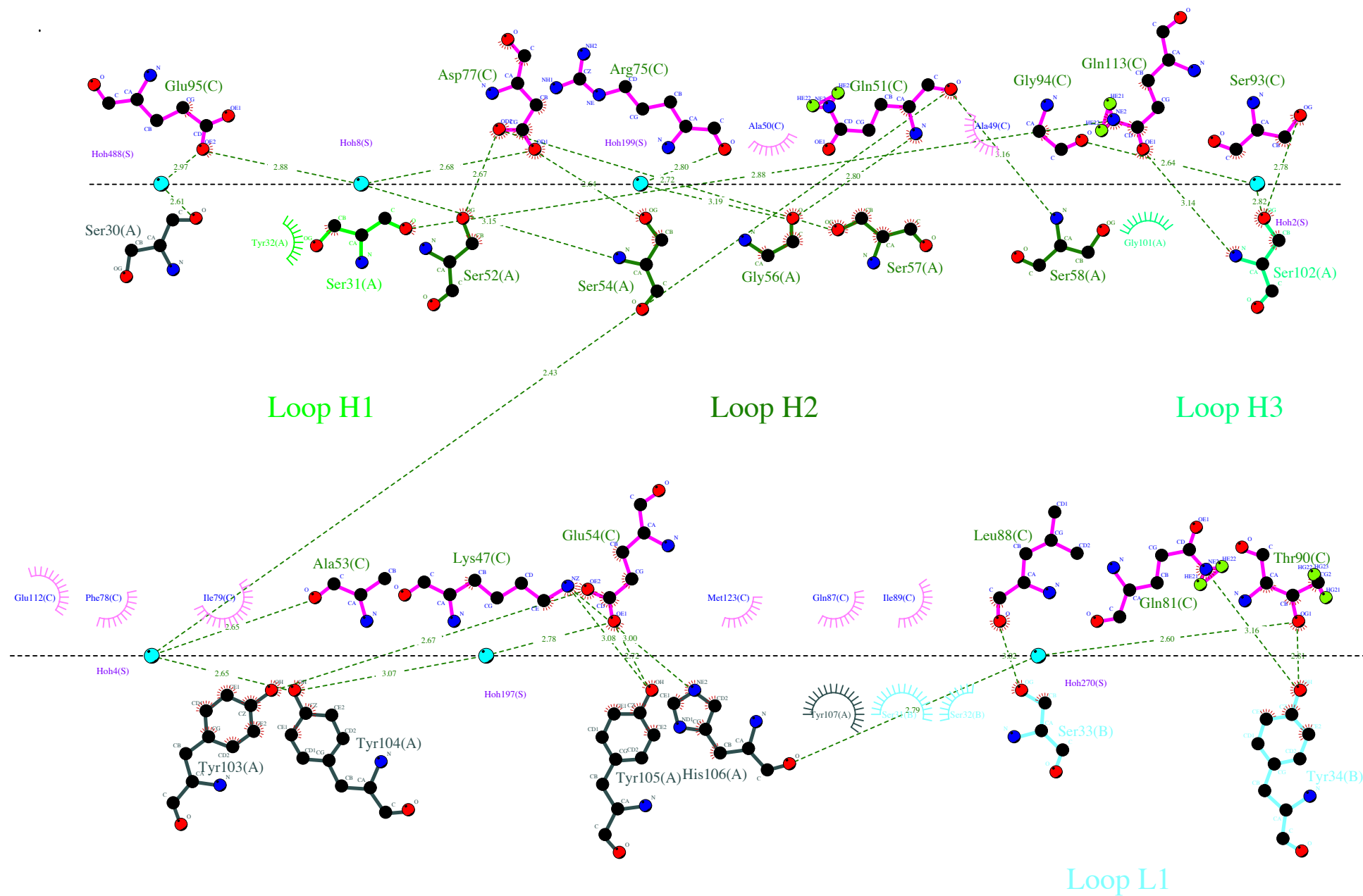

Supplement: S4 Fig — The bonds in the CDR loops of the heavy chain (chain A) are shown in different shades of green; bonds in the CDR-L1 loop of the light chain (B) are shown in aqua; bonds of the Fab outside of the CDR loops are shown in dark slate; bonds of FHbp are shown in magenta; and water molecules are shown in light blue. The Figure was generated using the Antibody feature in LigPlot+ and CDR loops were defined by Kabat convention implemented in LigPlot+ [56]. (PDF) [file ppat.1009655.s006.pdf]

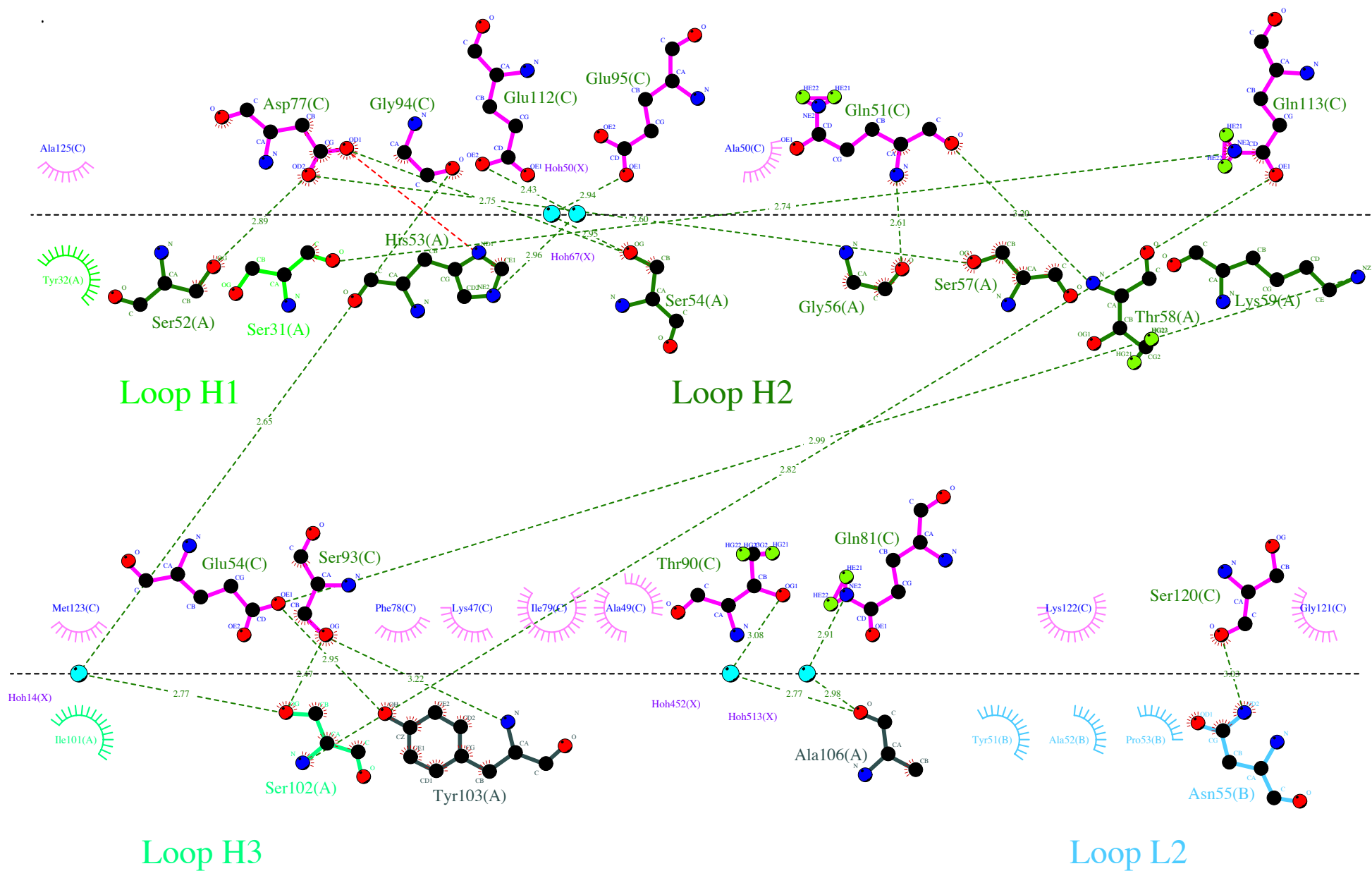

Supplement: S5 Fig — The color scheme is the same as in S4 Fig. The Figure was generated using the Antibody feature in LigPlot+ and CDR loops were defined by Kabat convention implemented in LigPlot+ [56]. (PDF) [file ppat.1009655.s007.pdf]

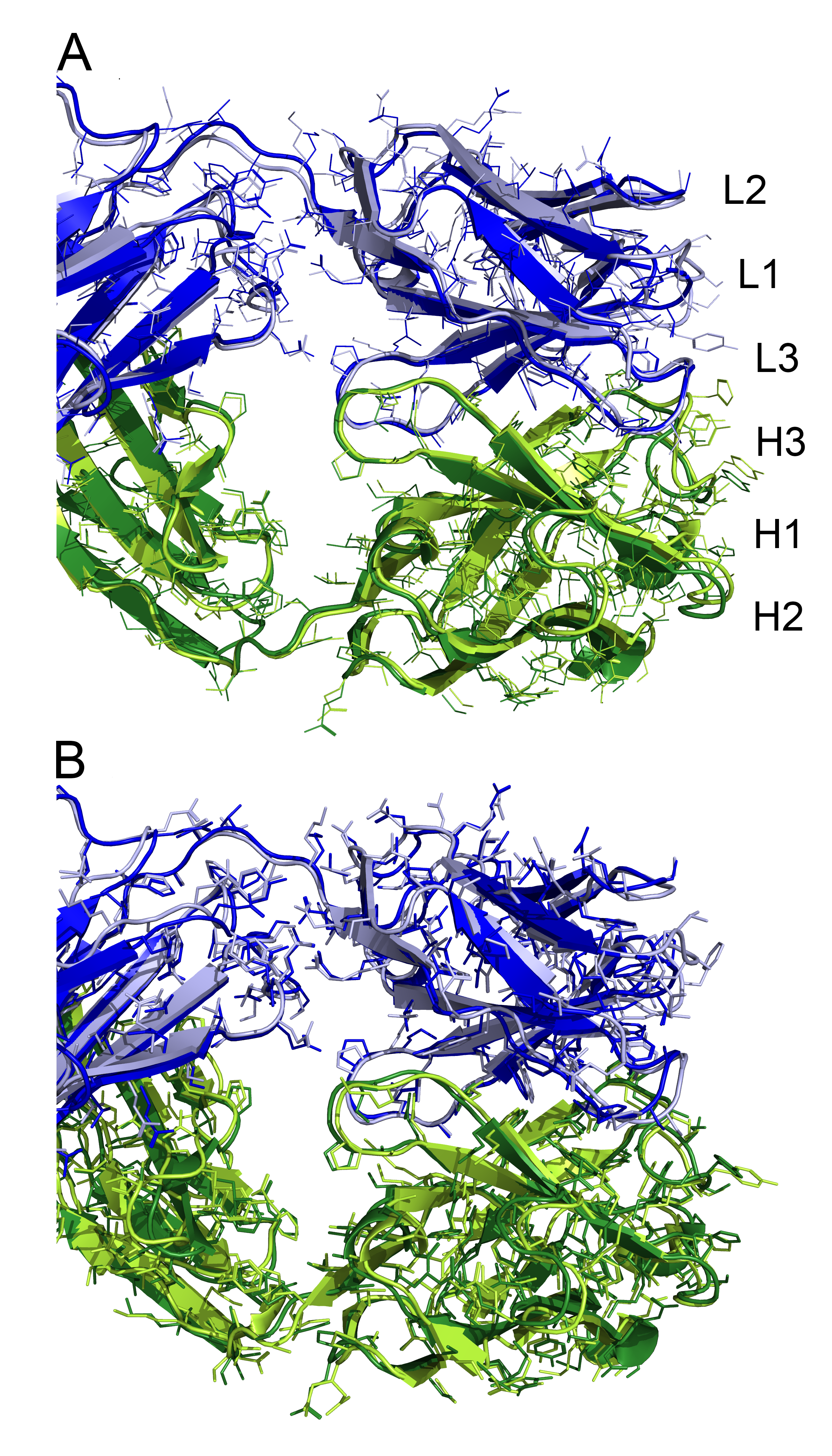

Supplement: S6 Fig — (A) FHbp-Fab 7B10 complex superimposed with FHbp-Fab 1A3 complex. (B) FHbp-Fab 7B10 complex superimposed with Fab 7B10 alone. For clarity, in both panels only the Fab molecules are shown. Figure generated with PyMol (Schrodinger, LLC). (TIF) [file ppat.1009655.s008.tif]

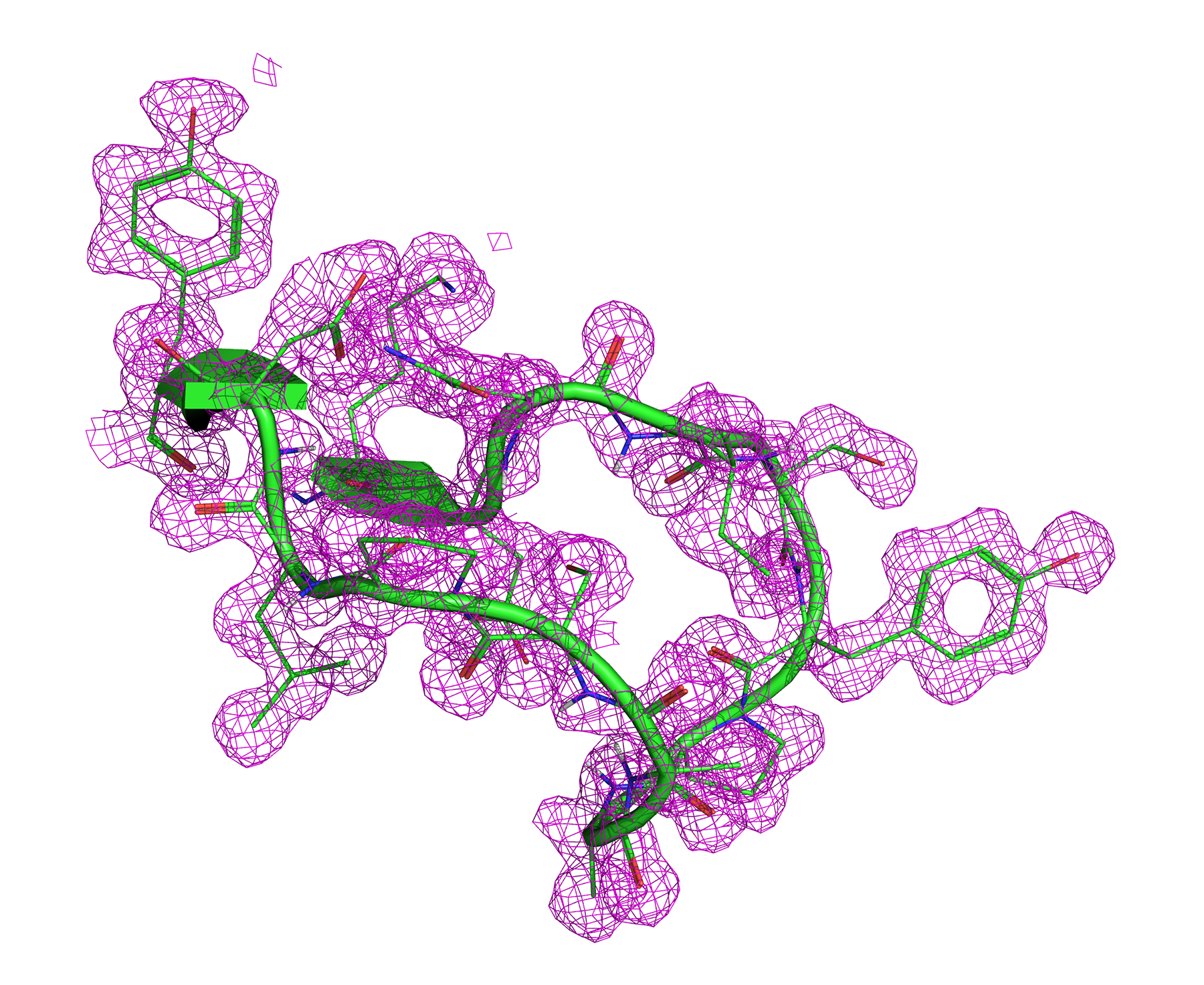

Supplement: S7 Fig — The 2Fo-Fc feature enhanced map [57] was calculated in Phenix [53] and the map is contoured at 1.2 sigma. Figure generated with PyMol (Schrodinger, LLC). (TIF) [file ppat.1009655.s009.tif]
