# Supplementary material for: Two human antibodies to a meningococcal serogroup B vaccine antigen enhance binding of complement Factor H by stabilizing the Factor H binding site
Source: PLoS Pathog. 2021 Jun 14;17(6):e1009655. doi: 10.1371/journal.ppat.1009655 (PMC8224966; doi:10.1371/journal.ppat.1009655)
Supplement: S3 Fig — (A) Heavy chain Fd fragment. (B) kappa light chains. The complementarity determining regions (CDR) of the heavy (H) and light (L) chains are shown in bold type. Accession numbers of the Fab sequences are given in Methods and alignment was performed with Clustal Omega [55]. (PDF) [file ppat.1009655.s005.pdf]

A

Human Heavy Chain Fd Fragment

```
                                    CDR-H1--->               CDR-H2----
Fab 1A3    EVQLVESGGGLVQPGGSLRLSCAASGFTFSSYAMSWVRQAPGKGLEWVSAISGSGGSSHY      60
Fab 7B10   QVQLVESGGGLVQPGGSLRLSCVTSGFTFRSYAMTWVRQAPGKGLEWVSSISHSGGSTKY      60
           :*********************.:***** ****:**************:** ****::*
           ------->                             CDR-H3------->
Fab 1A3    TDSVKGRFTISRDNSKNTLWLQMNSLRAEDTAIYYCAKTSGSYYYHYEIDVWGQGTTVTV     120
Fab 7B10   ADSVKGRLTISRDDSMDTLYLQMNSLRAEDTAIYYCAKDQISYPAASPLDYWGRGTLVTV     120
           :******:*****:* :**:****************** . **     :* **:** ***

Fab 1A3    SSASTKGPSVFPLAPSSKSTSGGTAALGCLVKDYFPEPVTVSWNSGALTSGVHTFPAVLQ     180
Fab 7B10   SSASTKGPSVFPLAPSSKSTSGGTAALGCLVKDYFPEPVTVSWNSGALTSGVHTFPAVLQ     180
           ************************************************************

Fab 1A3    SSGLYSLSSVVTVPSSSLGTQTYICNVNHKPSNTKVDKKVEPKSCDK                  227
Fab 7B10   SSGLYSLSSVVTVPSSSLGTQTYICNVNHKPSNTKVDKKVEPKSCDK                  227
           ***********************************************
```

B

Human Kappa Light Chain

```
                                  CDR-L1----->               CDR-L2>
Fab 1A3    EIVLTQSPGTLSLSPGERATLSCRASQSVSSSYLAWYQQKPGQAPRLLIYGTSNRATGIP      60
Fab 7B10   EIVLTQSPGTLSLSPGERATLSCRASQSFSSFSLAWYQQKPGQAPRLLIYAPSNRATGIA      60
           ****************************.**  *****************. *******
                                        CDR-L3-->
Fab 1A3    DRFSGSGSGTDFTLTVSRLEPEDFAVYYCQQYGSSPCSFGQGTKLELKRTVAAPSVFIFP     120
Fab 7B10   DRFSGSGSGTDFTLTISRLEPEDFAVYYCQQYGSSPITFGQGTRLEIKRTVAAPSVFIFP     120
           ***************:******************** :*****:**:*************

Fab 1A3    PSDEQLKSGTASVVCLLNNFYPREAKVQWKVDNALQSGNSQESVTEQDSKDSTYSLSSTL     180
Fab 7B10   PSDEQLKSGTASVVCLLNNFYPREAKVQWKVDNALQSGNSQESVTEQDSKDSTYSLSSTL     180
           ************************************************************

Fab 1A3    TLSKADYEKHKVYACEVTHQGLSSPVTKSFNRGECS                             216
Fab 7B10   TLSKADYEKHKLYACEVTHQGLSSPVTKSFNRGECS                             216
           ***********:************************
```
